# CT Texture Analysis in Breast Cancer Patients Undergoing CT-Guided Bone Biopsy: Correlations With Histopathology

**DOI:** 10.1177/11782234241305886

**Published:** 2025-01-29

**Authors:** Silvio Wermelskirchen, Jakob Leonhardi, Anne-Kathrin Höhn, Georg Osterhoff, Nikolas Schopow, Susanne Briest, Timm Denecke, Hans-Jonas Meyer

**Affiliations:** 1 Department of Diagnostic and Interventional Radiology, University of Leipzig, Leipzig, Germany; 2 Department of Pathology, University Hospital Leipzig, University of Leipzig, Leipzig, Germany; 3 Department of Orthopaedics, Trauma and Reconstructive Surgery, University Hospital Leipzig, Leipzig, Germany; 4 Department of Gynaecology, University Hospital Leipzig, Leipzig, Germany

**Keywords:** CT, bone biopsy, texture analysis

## Abstract

**Background:**

Texture analysis has the potential to deliver quantitative imaging markers. Patients receiving computed tomography (CT)-guided percutaneous bone biopsies could be characterized using texture analysis derived from CT. Especially for breast cancer (BC) patients, it could be crucial to better predict the outcome of the biopsy to better reflect the immunohistochemistry status of the tumor.

**Objectives:**

The present study examined the relationship between texture features and outcomes in patients with BC receiving CT-guided bone biopsies.

**Design:**

This study is based on a retrospective analysis.

**Methods:**

The present study included a total of 66 patients. All patients proceeded to undergo a CT-guided percutaneous bone biopsy, using an 11-gauge coaxial needle. Clinical and imaging characteristics as well as CT texture analysis were included in the analysis. Logistic regression analysis was performed to predict negative biopsy results.

**Results:**

Overall, 33 patients had osteolytic metastases (50%) and 33 had osteoblastic metastases (50%). The overall positivity rate for the biopsy was 75%. The clinical model exhibited a predictive accuracy for a positive biopsy result, as indicated by an area under the curve (AUC) of 0.73 [95% confidence interval (CI) = 0.63-0.83]. Several CT texture features were different between Luminal A and Luminal B cancers; the best discrimination was reached for “WavEnHH_s-3” with a *P*-value of .002. When comparing triple-negative to non–triple-negative cancers, several CT texture features were different, the best discrimination achieved “S(5,5)SumVarnc” with a *P*-value of .01. For the Her 2 discrimination, only 3 parameters reached statistical significance, “S(4,-4)SumOfSqs” with a *P*-value of .01.

**Conclusions:**

The utilization of CT texture features may facilitate a more accurate characterization of bone metastases in patients with BC. There is the potential to predict the immunohistochemical subtype with a high degree of accuracy. The identified parameters may prove useful in clinical decision-making and could help to identify patients at risk of a negative biopsy result.

## Introduction

Adequate histologic bone tissue sampling is of great clinical importance for oncological treatment and for the assessment of the metastatic situation in cancer patients.^1,2^

Computed tomography (CT)-guided percutaneous core needle biopsy (CNB) has widely been established as the standard for sampling and assessing suspected bone metastases.^3-6^ The CT guidance is beneficial in both reducing accidental trauma to adjacent organs and neurovascular structures, as well as in the confirmation of correct regional sampling.^7,8^

The existing literature presents conflicting findings regarding the diagnostic yield of CT-guided percutaneous CNB of suspected bone lesions, with estimated yields ranging between 69% and 87.4%,^4,5,9-12^ with the decision-making process being primarily dependent on the characteristics of the lesion in question, including the presence and degree of sclerosis, as well as the diameter of the lesion. Especially in breast cancer (BC) patients, a bone biopsy is routinely performed to confirm the metastatic stage and to obtain tissue for a histopathological determination of the immunohistochemically subtype. Correctly diagnosing the subtype is crucial, as chemotherapy regimen and additional endocrine hormone therapy can differ accordingly. Hence, guidelines recommend a biopsy at presentation and for the first recurrence of disease whenever possible.^12^

Especially BC patients can present with extensive sclerotic lesions in up to half of the patients, which can result in insufficient (negative) biopsy results. Interventions guided by cross-sectional imaging can improve the diagnostic yield of the biopsies.

Few investigations have addressed potentially influencing factors for diagnostic yield in BC patients like interventional or lesional characteristics.^4,5,11^ Nevertheless, most studies investigated distinct primary tumors undergoing CT-guided bone biopsies and were unable to ascertain whether BC patients might exhibit disparate characteristics. Furthermore, it is imperative to differentiate between osteolytic and osteoblastic metastases, as these have markedly dissimilar outcomes regarding CT-guided bone biopsies.^4,5,12^

With the emergence of texture analysis, novel biomarkers derived from radiological images can be used for additional quantitative image assessment.^13-17^ Computed tomography and magnetic resonance imaging (MRI) were used for texture analyses. Various texture analysis applications have been described in the literature, with its main focus being centered on further enhancing oncological decision-making.^13-17^ The utilization of disparate spatial characteristics facilitated more effective discrimination, treatment prediction, and prognosis stratification across a range of tumor entities. In essence, texture analysis derived from radiological images can provide quantitative information that extends beyond the scope of a radiologist’s clinical observation. This could be of significant clinical value in the further analysis of preinterventional images and the stratification of patients who may benefit from CT-guided intervention, as well as in the planning of biopsy regions. The application of quantitative imaging techniques should facilitate more informed clinical decision-making in patients with suspected bone metastases.

So far, no study has explored the diagnostic potential of texture analysis to predict the outcome of bone biopsies in BC patients.

Therefore, the aim of the present study was to investigate whether CT-derived texture analysis parameters can predict the histological bone biopsy result and possibly the immunohistochemical subtype of BC patients.

## Materials and Methods

### Patient acquisition

The present retrospective study has been approved by the institutional review board (Ethics Committee of the University of Leipzig, register no. 344-2007).

All patients undergoing a CT-guided bone biopsy from 01/2018 to 12/2022 were retrospectively assessed. Inclusion criteria were histologically confirmed primary BC, available CT imaging, availability of the histopathological results of the biopsy including the immunohistochemical subtype, and a detectable lesion on CT imaging. Exclusion criteria were severe imaging artifacts, eg, due to the presence of metal implants.

### Interventional procedure

Prior to the intervention, informed consent was obtained from each patient for the CT-guided biopsy. The biopsies were performed by radiologists with expertise in interventional radiology, with each having accrued over 5 years of experience. Before the procedure, complete blood count and coagulation profiles had been obtained. The biopsy was only performed in patients with a platelet count of at least 50.000/mm^3^, prothrombin time >50%, and partial thromboplastin time ⩽1.5 times. The alkaline phosphatase (AP) and lactate dehydrogenase (LDH) were extracted from the last blood sample before biopsy. All CT-guided bone biopsies were performed using the same 16-slice CT scanner (Brilliance Big Bore, Philips, Hamburg, Germany). The typical imaging parameters were as follows: 100 kVp; 125 mAs; slice thickness, 1 mm; pitch, 0.9. The CT scanner is described in further detail below. A conventional CT scan of the area of interest was conducted prior to the intervention, without the administration of contrast media. This scan was used to develop an optimal approach for accessing the lesion. Prior to the initiation of the procedure, the interventional strategy, particularly the patient’s position and biopsy pathway, was planned using prebiopsy CT images. The intervention was initiated with the administration of a topical antiseptic and the subsequent advancement of subcutaneous local anesthetic toward the planned biopsy pathway, with the delivery of 10 to 20 mL of lidocaine 1% (Xylocitin, Jenapharm, Germany). The biopsy was performed using a coaxial 11-gauge biopsy system. Subsequently, manual cortical penetration was performed, after which an automatic drill was employed to direct and penetrate the needle through the suspicious bone lesion. The periprocedural CT acquisitions were evaluated to assess whether the needle tip had reached the intended target lesion. Postbiopsy CT images were acquired for the purpose of detecting any potential complications, particularly hematoma.

### Conventional imaging analysis

An osteolytic metastasis was defined as a structural hypodense defect within the bone, characterized by a clear or irregular border. An osteoblastic metastasis was characterized as a hyperdense lesion of the bone, exhibiting a range of densities from slight hyperdense to strong hyperdense.

The length, angle, and Hounsfield units (HU) of the biopsy tract were measured on the preinterventional planning CT scan. The distance of the biopsy tract was determined by measuring the distance from the point of entry of the tract into the patient’s skin to the deepest penetration of the biopsy needle into the bone. In addition, the distance between the cortical entry of the biopsy needle to the deepest osseous penetration along the biopsied path was determined. Moreover, the HU of the osseous biopsy tract were determined by delineating a region of interest (ROI) on the preinterventional scan along the trajectory of the biopsy tract, as delineated by the subsequent interventional CT scans. The angle was determined by establishing a reference point at the deepest osseous penetration, with the biopsy tract serving as 1 arm of the angle and the shortest channel perpendicular to the plane of the skin serving as the other arm. An HU cut-off value of 610 HU was used as previously proposed by Donners et al.^18^

### Texture analysis

The CT images were analyzed with the texture analysis software MaZda (version 4.7, available at http://www.eletel.p.lodz.pl/mazda/).^19,20^ A polygonal ROI was delineated on the largest representative slide of the bone metastasis in the largest, proximal position. The ROI was then drawn in accordance with the following biopsy tract of the biopsy needle. The measurements were performed in a blinded manner, with the clinical results remaining unknown to the researchers. For each ROI, gray-level (µ) normalization was performed, using the limitation of dynamics to μ ± 3 standard deviations to minimize the influence of contrast and brightness variation, as it was performed in similar studies using texture analysis.^21^

**Figure 1.**
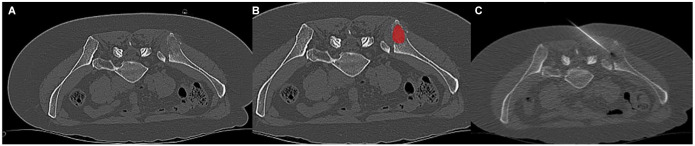
Representative case of the patient sample with osteolytic metastasis and triple-negative subtype. (A) The target lesion is located within the right iliac bone. The biopsy result was positive. (B) The region of interest is marked in red. (C) The biopsy needle confirmation within the lesion.

The extracted features were as follows: a gray-level histogram, a co-occurrence matrix, and a run-length matrix. The gray-level histogram included features such as contrast, angular second moment, correlation, entropy, sum average, sum entropy, sum variance, sum of squares, inverse difference moment, difference entropy, difference variance (for 4 directions), gray-level non-uniformity, run-length non-uniformity, short run emphasis, long run emphasis, and fraction of image in runs. The co-occurrence matrix included features such as sum of squares, sum average, correlation, and entropy. The run-length matrix included features such as gray-level non-uniformity, run-length non-uniformity, short run emphasis, long run emphasis, fraction of image in runs, and run-length variance. In addition, the extracted features included an absolute gradient, an autoregressive model (theta 1 to 4, sigma), and a wavelet transform. Moreover, 279 texture features were extracted for each patient. Figure 1 illustrates a representative case from the patient sample.

### Histopathology analysis

Once the biopsy samples had been obtained, they were fixed for a period of between 24 and 30 hours before undergoing a decalcification process involving ethylenediaminetetraacetic acid for a period of 48 hours. An experienced specialist pathologist evaluated the tumor content during the clinical work-up. The success of the histopathological examination was determined as either non-diagnostic or diagnostic, depending on whether a re-biopsy was required or a benign finding was observed following oncological follow-up. The patient groups were stratified according to the immunohistochemical subtype. Luminal A tumors were defined as those exhibiting positive staining for estrogen receptor (ER) or progesterone receptor (PR), negative for Her2, and low expression (<10%) of the proliferation marker Ki-67. Tumors designated as Luminal B were characterized by the presence of hormone receptors and a high expression of the proliferation marker Ki-67, with a percentage value exceeding 10%. The threshold values of 10% and 20%, respectively, were used to define high proliferative status.

The designation “Her2-enriched” was applied to cases where Her2 positivity was evident. A diagnosis of Her2-positivity was made only if the tumor scored 3+ on immunohistochemical staining or 2 with fluorescence in situ hybridization (FISH) positivity. Tumors were classified as triple-negative if they exhibited a lack of staining for both the ER and the PR, in addition to being Her2-negative.

The Ki-67 marker was stained using a polyclonal mouse antibody (clone Mib-1; DAKO/Agilent #M7240, Santa Clara, California) at 36°C for 32 minutes, with a dilution of 1:500 or 1:100. The Ki-67 proliferation index was determined by calculating the percentage of Ki-67-positive cells among the total number of tumor cells.

### Statistical analysis

The statistical analysis and graphical representation of the data were conducted using GraphPad Prism 8 (GraphPad Software, La Jolla, California) and SPSS (IBM, Version 25.0; Armonk, New York). The data were subjected to descriptive statistical analysis, including the calculation of absolute and relative frequencies. The Spearman correlation coefficient (r) was employed to analyze the associations between the investigated scores, following the testing of the data for normality of distribution. Group differences were calculated using the Mann-Whitney-U test and the Fisher’s exact test, as appropriate. A univariable and multivariable logistic regression analysis was employed to assess the relationships between the investigated parameters and immunohistochemistry subtype. The diagnostic accuracy was additionally investigated through the utilization of a receiver operating characteristics curve (ROC), with the area under the curve (AUC) being reported. A multivariate model was constructed in order to predict the optimal AUC. In all cases, a *P*-value of less than .05 was considered to indicate statistical significance. A power calculation was performed with the assumption of an incidence for a positive biopsy outcome of 80% in 1 subgroup and 65% in another subgroup, reflecting the published biopsy outcome range.^10,11^ The calculated sample size was 62 with a power of 0.80.

## Results

In total, 66 patients were included in the present study. The median age of the subjects at the time of CT acquisition was 64.7 years (interquartile range [IQR] = 22 years), with an age range of 43 to 84 years. The primary tumors were found to exhibit the following immunohistochemical subtypes: Twenty-six patients had Luminal A (53%), 15 Luminal B (31%), 12 were Her2 positive (24%), and 8 patients were triple-negative (16%).

A total of 38 lesions were identified within the pelvis (57.6%), followed by 24 lesions in the spine (36.4%) and 4 lesions within the sternum (6.1%). Of the lesions subjected to analysis, 28 patients exhibited osteolytic metastases (57.1%), whereas 21 patients displayed osteoblastic metastases (42.9%). The overall rate of tumor positivity in the biopsies was 75%. The clinical and imaging features are presented in Table 1 for reference. The positive biopsy rate was not statistically significantly different between osteolytic and osteoblastic metastases (85.3% vs 63.6%, *P* = .052). A further analysis was conducted to investigate the groups in relation to the biopsy results. In addition, the biopsy tract HU was found to be associated with the biopsy result. In the group of positive biopsies, the lesion HU was observed to be larger, with a mean value of 273.1 ± 233.5 mm, in comparison to the negative group, where the mean value was 19.32 ± 7.68 mm (*P* = .02). The mean HU of the lesion was observed to be lower in the positive biopsy group (281.44 ± 391.61 HU vs 481.29 ± 586; *P* = .02). In a further analysis, the groups were investigated regarding the biopsy results.

**Table 1.** Demographic overview of the patient sample.

Clinical and puncture feature	Biopsies (n = 66)
Osteolytic lesions N =, %	33 (50%)
Age (years)	64.61 ± 12.46
Lesion HU	332.92 ± 281.40
Longest lesion diameter	22.63 ± 10.62
Shortest lesion diameter	14.98 ± 7.26
Biopsy tract HU	309.61 ± 191.63
Biopsy tract length skin to lesion in mm	87.23 ± 21.60
Biopsy tract bone to lesion in mm	28.97 ± 15.03
Biopsy tract angle in °	32.27 ± 20.09
AP µkat/L	2.06 ± 1.56
LDH µkat/L	4.49 ± 0.95
Hb mmol/L	7.76 ± 1.00
Platelets exp 9/L	260.54 ± 79.40
Positive biopsies N =, %	49 (74%)
Her2(+) N =, %	12 (24%)
Her2(−) N =, %	37 (76%)
Ki-67 %	24.36 ± 20.95
Luminal A	26 (53%)
Luminal B	15 (31%)
Triple-negative	8 (16%)

The biopsy tract HU was also associated with the biopsy result. In the positive biopsy group, the lesion HU was larger with 273.1 ± 233.5 mm, whereas in the negative group, it was 19.32 ± 7.68 mm (*P* = .02). The mean HU of the lesion was lower in the positive biopsy group (281.44 ± 391.61 HU vs 481.29 ± 586; *P* = .02). Furthermore, AP significantly differs between the positive and negative biopsy cohorts. The AP values in positive biopsies showed significantly higher values of 2.3 ± 1.2 as in negative biopsies 1.38 ± 0.6. Table 2 presents the statistically significant texture features of the above-mentioned group comparisons. The histogram-based percentile parameters exhibited statistically significant differences in each of the group comparisons. Table 3 summarizes the statistically significant CT features according to the Ki-67 values of the tumors. The best discrimination reached “WavEnLH_s-4” for the 10% threshold value with a *P*-value of .005 and “WavEnHH_s-3” for the threshold value of 20% with a *P*-value of .01, respectively.

**Table 2.** Comparisons of the CT features accordingly to the biopsy result.

Imaging and texture features	Positive biopsy result	Negative biopsy result	*P*
_MinNorm	63.20 ± 28.69	91 ± 38.51	.01
_MaxNorm	145.47 ± 46.75	192.59 ± 61.31	.006
Mean	104.83 ± 30.33	142.27 ± 41.42	.002
Perc,01%	74.29 ± 26.17	101.71 ± 36.14	.02
Perc,10%	87.18 ± 26.84	120.47 ± 36.50	.002
Perc,50%	104.59 ± 30.93	142.41 ± 42 ± 42.04	.002
Perc,90%	122.53 ± 36.06	163.59 ± 48.37	.003
Perc,99%	136.55 ± 39.27	178.59 ± 52.32	.005
Teta2	−0.64 ± 0.14	−0.72 ± 0.12	.02
WavEnLL_s-3	17 767.45 ± 4000.52	14 352.31 ± 5251.06	.006
WavEnHL_s-3	333.30 ± 330.96	520.19 ± 334.29	.01
WavEnLL_s-2	17 314.85 ± 1928.62	16 435.02 ± 2773.73	.002

**Table 3.** Comparisons of the CT features accordingly to the Ki-67 proliferation index.

CT texture features	Ki-67 > 10%	Ki-67 < 10%	*P*	CT texture features	Ki-67 > 20%	Ki-67 < 20%	*P*
S(1,0)SumAverg	63.72 + 0.91	64.29 + 0.79	.01	S(0,5)SumVarnc	261.99 + 55.32	224.56 + 44.16	.02
S(1,-1)SumAverg	63.75 + 0.90	64.38 + 0.95	.006	WavEnHH_s-3	78.13 + 28.55	121.29 + 65.94	.01
S(2,0)SumAverg	63.69 + 1.10	64.37 + 1.07	.002				
S(2,-2)SumAverg	63.65 + 1.16	64.44 + 1.25	.02				
S(0,4)SumOfSqs	107.63 + 10.21	0.09 + 0.03	.002				
S(0,5)SumVarnc	251.27 + 56.59	220.70 + 38.24	.002				
WavEnHH_s-3	86.64 + 32.54	128.89 + 73.94	.003				
WavEnLH_s-4	232.83 + 281.28	318.79 + 206.44	.005				

Table 4 provides the statistically significant CT features for the comparisons of the different immunohistochemically subtypes. Several CT texture features were different between Luminal A and Luminal B cancers; the best discrimination was reached for “WavEnHH_s-3” with a *P*-value of .002.

**Table 4.** Comparisons of the CT features accordingly to the immunohistochemical subtype.

CT texture features	Luminal A	Luminal B	*P*
Biopsy tract HU	120.69 ± 41.64	95.85 ± 28.96	.02
S(3,-3)SumOfSqs	108.66 ± 8.61	101.04 ± 9.47	.02
S(4,0)SumOfSqs	107.93 ± 9.12	101.28 ± 8.91	.02
S(4,-4)SumOfSqs	111.70 ± 12.12	102.03 ± 13.51	.01
S(4,-4)SumAverg	64.24 ± 1.47	63.21 ± 1.02	.04
S(5,0)SumOfSqs	109.26 ± 10.97	101.85 ± 9.14	.02
S(5,5)SumOfSqs	105.23 ± 12.61	92.78 ± 24.72	.04
WavEnHH_s-3	127.83 ± 67.69	73.35 ± 26.75	.002
CT texture features	Triple negative	Non-triple negative	*P*
S(1,1)SumOfSqs	110.76 ± 5.21	0.62 ± 0.27	.04
S(3,3)SumVarnc	296.18 ± 66.52	64.12 ± 1.45	.03
S(4,0)SumVarnc	306.62 ± 68.61	64.05 ± 1.36	.04
S(4,4)Correlat	0.30 ± 0.22	182.13 ± 59.13	.02
S(4,4)SumVarnc	286.93 ± 56.93	64.21 ± 1.88	.02
S(5,0)Correlat	0.30 ± 0.24	193.66 ± 63.04	.02
S(5,0)SumVarnc	293.87 ± 63.12	64.07 ± 1.44	.02
S(0,5)Correlat	0.32 ± 0.24	184.86 ± 58.74	.04
S(0,5)SumVarnc	281.89 ± 49.92	63.95 ± 1.75	.02
S(5,5)Correlat	0.26 ± 0.23	189.45 ± 58.48	.02
S(5,5)SumVarnc	275.58 ± 56.31	64.07 ± 2.48	.01
WavEnHH_s-4	203.18 ± 107.36	475.91 ± 517.89	.04
CT texture features	Her2(+)	Her2(−)	*P*
S(4,0)SumOfSqs	102.54 ± 8.01	108.09 ± 101.01	.04
S(4,-4)SumOfSqs	100.62 ± 11.33	110.95 ± 12.65	.01
WavEnHH_s-3	74.12 ± 27.54	117.97 ± 63.18	.02

For the comparison between triple-negative and non–triple-negative cancers, several CT texture features were different, the best result achieved “S(5,5)SumVarnc” with a *P*-value of .01. For the Her 2 discrimination, only 3 parameters reached statistically significance, “S(4,-4)SumOfSqs” with a *P*-value of .01.

### Diagnostic accuracy of texture features for the prediction of histopathological subtypes

**Figure 2.**
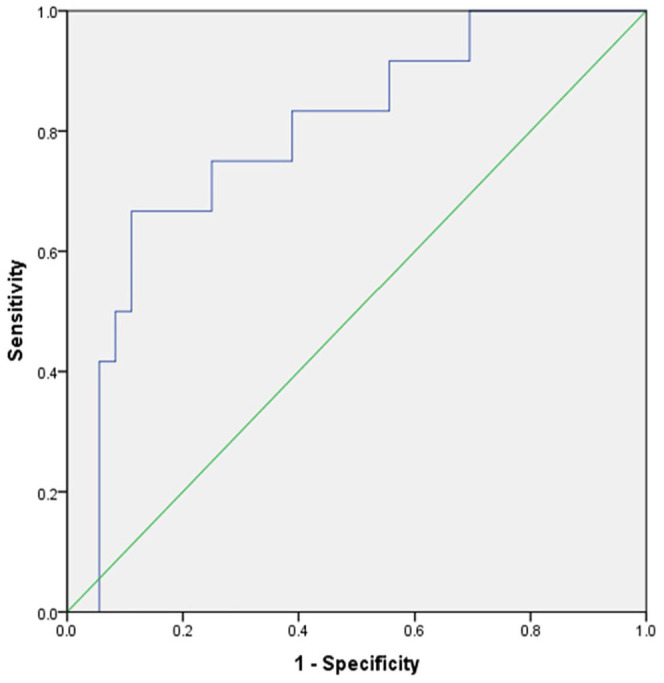
The CT texture multivariate logistic regression model regarding the Her2 status. The developed model yielded an AUC of 0.79 [95% CI = 0.65-0.94], resulting in an optimal sensitivity and specificity of both 0.75.

The relationships between the significant texture features (n = 4) and the positive or negative Her2 state were analyzed using multivariate logistic regression. The developed model yielded an AUC of 0.79 [95% confidence interval (CI) = 0.65-0.94], resulting in an optimal sensitivity and specificity of 0.75. The corresponding graph is displayed in Figure 2.

**Figure 3.**
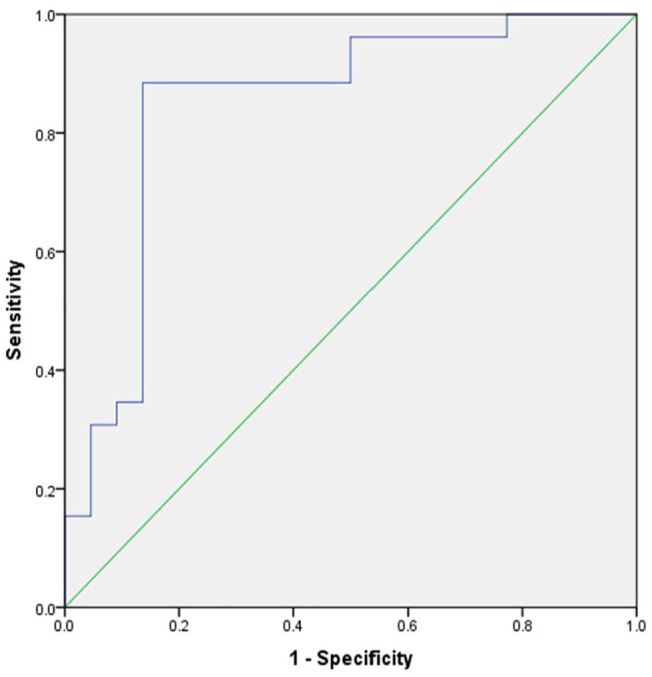
The CT texture feature multivariate model for Luminal A type demonstrated a prediction accuracy with an AUC of 0.85 [95% CI: 0.73-0.97]. The resulting optimal sensitivity and specificity are 0.89 and 0.86, respectively.

Furthermore, multivariate logistic regression was employed to examine the correlation between the 8 significant texture features and positive or negative Luminal A and Luminal B. The model for Luminal A exhibited a high level of prediction accuracy, with an AUC of 0.85 [95% CI = 0.73-0.97] (Figure 3). The resulting optimal sensitivity and specificity are 0.89 and 0.86, respectively.

**Figure 4.**
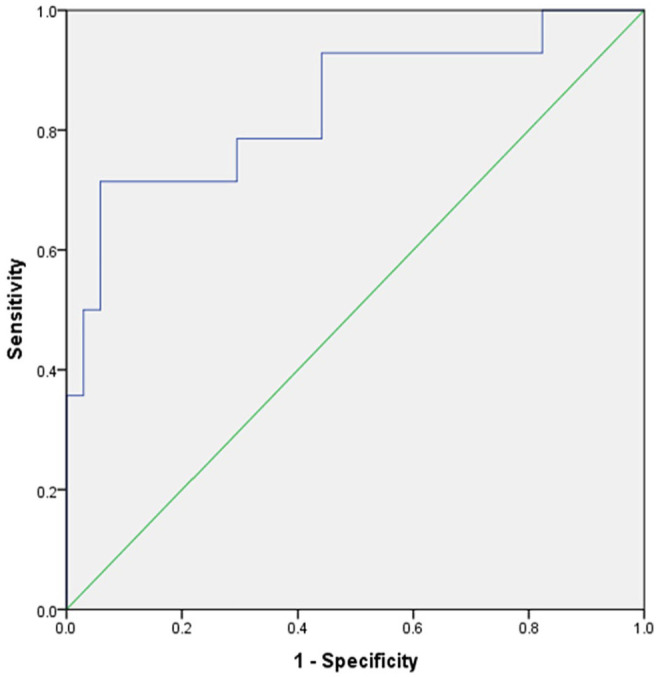
The prediction for Luminal B type demonstrated a prediction accuracy with an AUC of 0.84 [95% CI = 0.70-0.98], leading to a sensitivity of 0.79 and a specificity 0.71.

The model for Luminal B demonstrated a prediction accuracy with an AUC of 0.84 [95% CI = 0.70-0.98] (Figure 4), resulting in a sensitivity of 0.79 and a specificity of 0.71 when an optimal threshold was applied.

**Figure 5.**
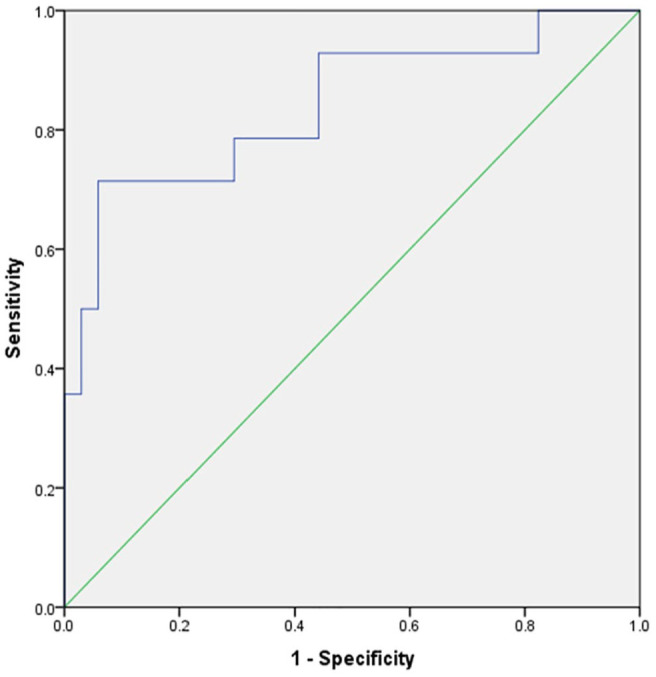
The multivariate prediction for triple-negative type showed an accuracy of 1.00 [95% CI = 1.00-1.00], which results in a sensitivity and specificity of both 1.00, respectively.

Similarly, a multivariate logistic regression analysis was conducted to evaluate the correlation between the significant texture features (n = 15) and the presence of triple-negative BC. The model exhibited superior prediction accuracy, with an AUC of 1.00 [95% CI = 1.00-1.00] (Figure 5), resulting in a sensitivity and specificity of 1.00.

## Discussion

The present study evaluated the diagnostic benefit of CT texture analysis and clinical parameters in predicting the outcome of CT-guided osseous biopsies in patients with BC.

It was demonstrated that the CT texture features of the target lesion vary in accordance with the biopsy outcome. This could facilitate more effective clinical work-up, enabling more accurate patient stratification and the selection of the optimal biopsy target, thereby enhancing patient care. Furthermore, it was demonstrated that CT texture features may be capable of predicting the immunohistochemical subtype of BC.

The present results could be used to better guide the interventional radiologist to better choose the target lesion. In the event that a patient presents with multiple lesions, it is recommended that the CT texture analysis be integrated into the decision-making process to determine which lesion should be biopsied, with the aim of improving the biopsy outcome rate. There is a definite need for a prospective evaluation of our results in further studies.

According to the published guidelines of the European Society for Medical Oncology, biopsies of bone metastases should be avoided, whenever possible, due to the limited possibilities of biomarker detection in decalcified tissue and therefore poorer diagnostic yield of the biopsy.^22^

Only a small amount of data have been published to examine the potential associations between imaging and clinical data with the diagnostic yield of bone biopsies in patients with BC. A recent study investigated bone biopsies of 56 BC patients and reported a high sensitivity of 93.6%, a specificity of 100%, and overall accuracy of 94.8% for bone biopsies in patients with BC.^23^ Another finding of this study was that sclerotic lesions had poorer overall sample quality compared with lytic lesions.^23^

We could demonstrate different results stratified accordingly to lesion type and immunohistochemical subtype in patients with BC. This is especially of importance, as it was demonstrated before that the appearance of bone metastases in BC differ depending on its histological subtype.^24^ This could be one reason why we were able to demonstrate a good accuracy of CT texture analysis to predict the immunohistochemical subtype.

As reported previously, there was a slight discordance between the primary tumor and the hormone receptor status in about 33.3% of cases, whereas the Her 2 status was almost concordant in every case.^23^

The identified differences of CT texture analysis, accordingly to receptor status, might be explained by the known fact that the metastasis pattern is highly correlated with the immunohistochemical subtype.^24,25^ Presumably, CT texture is able to characterize the microstructure of tissues and might be able to identify the molecular and cellular differences in the microenvironment of different immunohistochemical subtypes.^26^ This needs to be confirmed in experimental analyses.

To overcome these identified shortcomings of bone biopsies, texture analysis could enable a better tissue characterization based on spatial differences of the CT images.^13-17,27^ It has been shown before that CT texture analysis could provide insights into the biological behavior of tumors.^13-17,27^ In a study on head and neck cancer patients, the CT texture parameter entropy, a marker for the heterogeneity of the tumor, was directly associated with the hypoxia-inducible factor-1 demonstrating the direct link between histology and CT texture analysis.^10^ In a similar way, CT texture analysis was able to predict histological subtypes of lung cancer patients and showed significant associations with P63 and P40 expressions.^28^ The present results should be interpreted in a similar way that CT texture features are correlated with the underlying histopathology and are able to characterize the tumors.

One of the principal findings of our study is that the conventional imaging markers, including biopsy length, HU of the lesion, and lesion diameter, are inadequate for accurately predicting biopsy outcomes in BC patients. In comparison, quantitative CT texture analysis is a more reliable predictor of biopsy outcomes.

It is clear that images can harbor more information than are currently extracted. The overall tumor-positive biopsy rate of 75% in our study is within the pooled frequency of 62% to 82%, which was published in a meta-analysis of 13 studies analyzing only sclerotic bone biopsies.^25^ This emphasizes the importance that osteoblastic lesions should be avoided for tissue sampling—when clinically possible. The present higher positivity rate for osteolytic lesions compared with sclerotic lesions is a common finding in the literature.^10,26^

The current findings indicate that further investigation of CT texture analysis is warranted in a prospective study design that incorporates a range of interventional procedures and aims to use CT texture analysis for preliminary prediction of the immunohistochemical subtype.

Presumably, CT texture analysis could also be used in patients with no possible invasive biopsy due to bleeding risk or non-reachable target localization to provide insight into the immunohistochemical subtype. The present results suggest that the identified diagnostic accuracy in our cohort might be high enough to provide a good surrogate prediction by imaging. There is a definite need for a validation study in those patients to see whether CT texture analysis could enhance the clinical workflow in this clinical situation.

It should be noted that the present study is not without limitations. First, it is a retrospective study, which may be susceptible to inherent bias. Second, the sample size of patients is relatively limited, which has resulted in a small number of subgroups and, subsequently, small subgroups for the immunohistochemical types. This may also result in the overfitting of our prediction models. The high diagnostic accuracies, particularly in the triple-negative subgroup analysis, should be interpreted with caution and require further validation in an external patient cohort. Third, although an 11G needle was used in all cases, slight variations in needle length and extracted bone biopsy could have influenced the biopsy result. Fourth, due to the limited sample size, it was not feasible to divide the patient cohort into a training and validation cohort, which may have reduced the external validity of the present results.

## Conclusions

The present study demonstrated that CT texture features have the potential to characterize bone metastasis in patients with BCs and to predict the immunohistochemical subtype. Inclusion of CT texture analysis in clinical decision-making could facilitate the identification of patients at risk of a negative biopsy result.

## Declarations

**Ethics approval and consent to participate:** This retrospective study was approved by the institutional review board (University of Leipzig, register no. 344-2007). Informed consent was waived due to the retrospective design. Written informed consent from participants was not required in accordance with local guidelines.

**Consent for publication:** Not applicable.

**Availability of data and materials:** The data sets used and/or analyzed during the current study are available from the corresponding author on reasonable request.
